# Triggered Escalating Real-Time Adherence Intervention to Promote Rapid HIV Viral Suppression Among Youth Living With HIV Failing Antiretroviral Therapy: Protocol for a Triggered Escalating Real-Time Adherence Intervention

**DOI:** 10.2196/11416

**Published:** 2019-03-18

**Authors:** K Rivet Amico, Amanda Dunlap, Ronald Dallas, Jane Lindsey, Barbara Heckman, Patricia Flynn, Sonia Lee, Keith Horvath, Rachel West Goolsby, Michael Hudgens, Teresa Filipowicz, Melissa Polier, Emily Hill, Megan Mueller Johnson, Jessica Miller, Anne Neilan, Andrea Ciaranello, Aditya Gaur

**Affiliations:** 1 School of Public Health Department of Health Behavior and Health Education University of Michigan Ann Arbor, MI United States; 2 St. Jude Children’s Research Hospital Department of Infectious Diseases Memphis, TN United States; 3 Chan School of Public Health Department of Biostatistics Harvard University Boston, MA United States; 4 Frontier Science and Technology Research Foundation Amherst, NY United States; 5 National Institutes of Health Eunice Kennedy Shriver National Institute of Child Health and Human Development Bethesda, MD United States; 6 School of Public Health Division of Epidemiology and Community Health University of Minnesota Minneapolis, MN United States; 7 Gillings School of Public Health Department of Biostatistics, Collaborative Studies Coordinating Center University of North Carolina at Chapel Hill Chapel Hill, NC United States; 8 Massachusetts General Hospital Division of Infectious Diseases Boston, MA United States

**Keywords:** HIV, adolescents, medication adherence, telemedicine

## Abstract

**Background:**

Youth living with HIV (YLWH) are confronted with many self-care challenges that can be experienced as overwhelming in the context of normal developmental processes that characterize adolescence and young adulthood. A sizable minority of YLWH have unsuppressed viral loads in the United States attributable to antiretroviral therapy (ART) nonadherence. Interventions to promote sustained viral suppression in YLWH are needed.

**Objective:**

The aim of this study is to evaluate the efficacy of the Triggered Escalating Real-Time Adherence (TERA) intervention in comparison with standard of care (SOC) in YLWH (aged 13-24 years) failing ART on (1) primary outcome measures—HIV viral suppression (VLS), defined as both <200 copies/ml and <50 copies/ml at 12 weeks, and (2) secondary outcome measures—VLS rates and rates of ART adherence at 24, 36, and 48 weeks as well as patterns of adherence over time as measured by an electronic dose monitoring (EDM) device.

**Methods:**

The TERA study is a phase 2, multisite clinical trial conducted with 120 YLWH failing ART (randomized 1:1 to TERA or SOC) at participating clinical sites within the Adolescent Medicine Trials Network for HIV/AIDS Interventions (ATN). Participants are followed for a total of 48 weeks. For TERA arm participants, the first 12 weeks involve delivery of the intervention. For all participants, clinical outcomes are collected throughout follow-up, and adherence is assessed using EDM over the full 48 weeks. During the 12-week intervention period, TERA arm participants receive 3 remote coaching sessions delivered in clinic via videoconferencing timed to coincide with baseline and follow-up clinical visits, text message reminders when the EDM has not been opened at dose time (which escalate to 2-way theory-informed short message service coaching interactions in response to real-time nonadherence), and review of dosing graphs produced by EDM at follow-up visits.

**Results:**

Launch dates for enrollment varied by site. Enrollment began in April 2018 and is expected to be completed by August 2019, with results presented by the second quarter of 2021.

**Conclusions:**

Effective, generalizable, and scalable approaches to rapidly assist YLWH failing to achieve and sustain VLS may have a substantial impact on individual health and efforts to curb transmission. Coaching for a brief but intensive period from remote coaches and using communication channels common to youth may offer multiple unique advantages in promoting self-care.

**Trial Registration:**

ClinicalTrials.gov NCT03292432; https://clinicaltrials.gov/ct2/show/NCT03292432 (Archived by WebCite at http://www.webcitation.org/768J8ijjp).

**International Registered Report Identifier (IRRID):**

DERR1-10.2196/11416

## Introduction

### Background

Among the 35 million people living with HIV worldwide, 7 million are below the age of 24 years. With the concerted efforts to increase viral suppression globally and through strategic national plans, the unique demands and challenges facing youth living with HIV (YLWH) must be addressed to reach viral suppression goals. Successful progression through the continuum of HIV care is poorer among adolescents than adults in the United States, with as many as 46% of those started on antiretroviral therapy (ART) not achieving viral suppression (VLS) in large part due to difficulties with medication adherence [1]. Recent reviews suggest that the subset of youth who achieve VLS after 48 weeks of ART can be alarmingly low (27% to 89%) [2]. Sustaining VLS is critical both for the individual health and to curb transmission. As those failing ART for adherence reasons are at an increased risk of subsequent ART failure [3,4] and problems with adherence appear to be relatively common [5,6], interventions to improve adherence and disrupt patterns of nonadherence are critically needed.

A dearth of evidence exists on the issues, approaches, and facilitators/barriers for YLWH failing to achieve or sustain VLS [7,8]. Literature specific to adults failing first-line ART suggests that sex (female) and delayed start of second-line therapy predict lack of suppression by 24 weeks [9], and although the evidence base in characterizing first-line ART failures and second-line outcomes in resource-limited settings is growing [10], this may not generalize to YLWH in the United States.

YLWH between the ages of 13 and 24 years are a unique cohort. Developmental tasks during this period of life create both challenges and resources [11] that impact daily living. Youth would likely benefit most from strategies that specifically bridge the gaps common during adolescence caused by normal neuro-cognitive and emotional development [12]. Brain maturation in the limbic system and prefrontal cortex, amongst other changing hormonal and environmental dynamics, can be expressed in poor decision-making, impulsivity, lower self-care, and higher engagement in risk behavior [13]. Challenges in executive functioning and cognitive abilities that limit abilities to plan, organize, focus attention, or manage rewards have been identified in HIV-infected youth, even before ART initiation [14]. Psychologically, identity development tasks heighten awareness and sensitivity to belonging and fitting in, which may exacerbate feelings of stigma among youth negotiating adherence [15]. Support for YLWH struggling with adherence must extend beyond and in-between clinical care visits, which can be achieved through technology-based modalities. Interventions that offer continuous contact and implement as-and-when-needed outreach may be well matched to the dynamics of everyday life for youth in the United States. Furthermore, with most youth also using cell phones in the United States, delivering support between visits can be facilitated through texting. The Triggered Escalating Real-Time Adherence (TERA) study (the Adolescent Medicine Trials Network for HIV/AIDS Interventions [ATN] 152) seeks to evaluate an intervention package that leverages several of these components: real-time electronic dose monitoring (EDM) that signals potential intervention opportunities, texting to explore needs and context surrounding such events, and phone-delivered outreach to offer patient-centered coaching from remotely located coaches.

### Rationale for Triggered Escalating Real-Time Adherence Intervention

The rationale for each of the active components of the TERA intervention (coaching, EDM, short message service [SMS], phone-based outreach, and time-limited with remote placement of coaches) is presented separately below, whereas the manner in which TERA combines these components is provided in the description of the implementation of the intervention.

Coaching has been defined as a patient-centered, strength-based approach that tends to be time-limited, focused on problem-solving and health and wellness, and goal oriented, which differs from counseling predominantly in its explicit focus on current lifestyle and more narrow scope and depth [16]. Coaches tend to provide when and as needed support and check-ins [16], and the approach has gained support in addressing diverse health behaviors, including weight management, exercise, and overall physical health [17]. In a review of coaching interventions, approaches that included goal setting and motivational interviewing demonstrated stronger outcomes [18]. Despite the promise for marginalized groups [19] and for adolescents [20], coaching work to date has largely focused on adults. TERA will be one of the first studies to evaluate coaching tied to EDM monitoring for youth struggling with ART adherence.

An additional core component of the TERA intervention package is the use of EDM. Evidence supports the use of technology-enhanced interventions to promote medication adherence among those living with HIV [21]. A recent study does, however, caution that reminders tethered to EDM identified that missed doses may lack impact. A study with adults in Cape Town, South Africa, evaluated an intervention that used SMS text messages to signal late doses according to a Wisepill device for first-line therapy ART [22]. Although a slight reduction in treatment interruptions was reported, overall adherence, retention in care, and VLS did not improve, which may have been due to reliance on “one-way” texting. One-way texting (with no expectation or opportunity to reply or engage in a conversation) appears to have an inconsistent impact on adherence, whereas interactive texting (where replies are expected and conversation is possible) has consistent support [23-25].

SMS text-based strategies [26,27], which are not yoked to monitoring but are systematically sent, require a response from participants and also seem to suggest that an interactive component is particularly beneficial [28,29]. Garofalo et al’s recent study in this area with 105 adolescents and young adults where 2-way daily SMS texts were provided to nonadherent youth demonstrated a significant improvement in self-reported adherence and high satisfaction scores [30]. Furthermore, a pilot study using personalized, interactive, daily SMS text messages demonstrated significant improvement among 14- to 29-year-olds living with HIV with poor self-reported adherence rates [31]. Coupled with consistent support for text-based “well-checks,” where patients receive an interactive text weekly simply asking how they are doing [26,27], using SMS interactive texting with a population who uses texting as part of the fabric of their daily lives was identified as a critical component to incorporate in the TERA intervention. For the TERA intervention, texting with patients about an EDM-identified late dose or simply checking in on their well-being had a strong basis for a potentially effective avenue of communication between coaches and participants. Finally, another promising aspect of collecting EDM data is the use of these data to present dosing patterns to patients and offer opportunities to discuss, problem solve, and reflect on adherence patterns. Previous research using this strategy with adults has demonstrated an impact on adherence [32,33] and could be appropriate for youth as well.

Moreover, related to EDM-identified late dosing, phone-based follow-up was identified as a promising strategy to connect with youth when and as needed. Evidence has supported the utility of phone-based outreach using a problem-solving approach [34,35]. In a recent review, harnessing mobile phone technology was identified as a promising area for future interventions encouraging optimal adherence among YLWH [30]. Furthermore, evidence suggests that using phone-based technology to engage adolescent social support networks may promote optimal engagement in care and adherence to medications [36,37]. A recent study of a phone-based support intervention among nonadherent YLWH found that it was acceptable and feasible among youth and clinic staff [38].

Finally, the rationale for using remote coaches and a time-limited intervention reflects efforts to develop interventions that are generalizable and can be brought to scale if effective. Despite the urgent need for services to assist youth failing ART to reach and sustain viral suppression, most clinics in the United States report this group as a small portion of the patient population. Thus, even if intensive interventions such as TERA are effective in improving disease outcomes, large investments to hire a full-time coach or offer dose monitoring off-hours and on weekends will have limited return for clinical care sites at the patient population level. Locating coaches remotely allows a single coach to work remotely and intensively with identified youth from multiple clinics across the United States. The TERA intervention brings the intervention to those who could benefit from it, rather than having patients physically present themselves to a specific location to receive support. In addition, because the approach is intensive, limiting its implementation to a discrete period is intended to optimize feasibility and acceptability of the approach. If effective, TERA could be developed as an independent service, available for youth across the United States, prescribed by providers and potentially covered by insurance. Together, remote and time-limited features of the intervention are intended to facilitate the viability and speed of scale-up.

### Study Objectives

To support YLWH failing ART due to nonadherence, the TERA study will evaluate a novel, evidence-informed, triggered, escalating, real-time adherence intervention among 120 youth with unsuppressed virus. The study objectives and hypotheses are listed in Table 1.

### 

**Table 1:** Objectives, outcomes, and hypotheses.

Level	Objective^a^	Measure(s)	Hypothesis
Primary objective #1a	To estimate and compare HIV viral suppression rates in YLWH^b^ 12 weeks after initiating TERA^c^ or continuing SOC^d^	HIV-1 RNA <50 copies/ml	Youth in the TERA arm will be more likely to achieve viral suppression (VLS) at week 12 compared with youth in the SOC arm^e^
Primary objective #1b	To estimate and compare HIV viral suppression rates in YLWH 12 weeks after initiating TERA or continuing SOC	HIV-1 RNA <200 copies/ml	Youth in the TERA arm will be more likely to achieve VLS at week 12 compared with youth in the SOC arm^a^
Secondary objective #1	To estimate and compare viral suppression rates in YLWH 24, 36, and 48 weeks after initiating TERA or continuing SOC	HIV-1 RNA <50 copies/ml and HIV-1 RNA <200 copies/ml	Youth in the TERA arm will be more likely to achieve VLS at weeks 24, 36 and 48 compared with youth in the SOC arm^f^
Secondary objective #2	To estimate and compare proportions of participants initiating TERA or continuing SOC who achieve viral suppression (HIV-1 RNA <200 copies/ml) by 12 weeks and maintain viral suppression through 48 weeks	HIV-1 RNA <200 copies/ml at weeks 12, 24, 36, and 48	Youth in the TERA arm will be more likely to achieve and sustain VLS than those in the SOC arm^g^
Secondary objective #3	To summarize and compare adherence patterns in YLWH initiating TERA or continuing SOC during the intervention period (weeks 0-12) and the postintervention period (weeks 12-48)	EDM^h^ on-time adherence and nonpersistence (between week 0-12, 12-24, 24-36, and 36-48)	Youth in the TERA arm will have higher rates of weekly dosing as measured by EDM over 48 weeks than those in the SOC arm

^a^Other and exploratory objectives, which focus on social psychological changes over time and between arms, classification of patterns of adherence per EDM data, mixed methods characterization of acceptability and feasibility of the TERA intervention and study participants, and costing data, are also included in the protocol.

^b^YLWH: youth living with HIV.

^c^TERA: Triggered Escalating Real-Time Adherence.

^d^SOC: standard of care

^e^Participants with no HIV-1 RNA measurement within the allocated week 12 study visit window (± 14 days) will be classified as failures.

^f^Participants with no HIV-1 RNA measurement within the allocated study visit window for weeks 24, 36, or 48 (±28 days) will be classified as failures.

^g^Participants will be classified as virologic successes if both the week 12 (±14 days) and week 48 (±28 days) HIV-1 RNA measurements are <200 copies/ml and at least one of the week 24 (± 28 days) or week 36 (± 28 days) HIV-1 RNA measurements is <200 copies/ml.

^h^EDM: electronic dose monitoring.

The primary objective, 12-week viral suppression, will be measured at the week 12 visit when the final coaching session occurs, thus representing the effects at the end of the intervention. Both the US Department of Health and Human Services viral load cutoff of 200 copies/ml used to define virologic failure [39] as well as the cutoff of 50 copies/ml increasingly used in research studies to define undetectable HIV viral load are used to describe the viral load–based primary and secondary outcome measures. Note that our use of both a stringent definition of VLS (<50 copies/ml) as well as a less restrictive but well-recognized cutoff of <200 copies/ml reflects evidence suggesting that each criterion may offer unique insights into control of virus, risk for viral rebound, and potential for development of resistance [40] and is consistent with the most recent Antiretroviral Guidelines for Adults and Adolescents [39]. The secondary objectives include viral suppression at subsequent time points to evaluate the longitudinal efficacy of the coaching sessions on viral suppression, and similarly use the <50 copies/ml as well as the <200 copies/ml cutoffs at weeks 24, 36, and 48, and the “durability” of intervention effects through characterizing the percentage of youth in each arm who achieved and maintained each operationalization of viral suppression. Finally, collected EDM data will be explored to determine whether EDM-identified adherence through week 12 and from weeks 12 to 48 was better in the intervention condition.

### Theoretical Basis for Intervention

**Figure 1:**
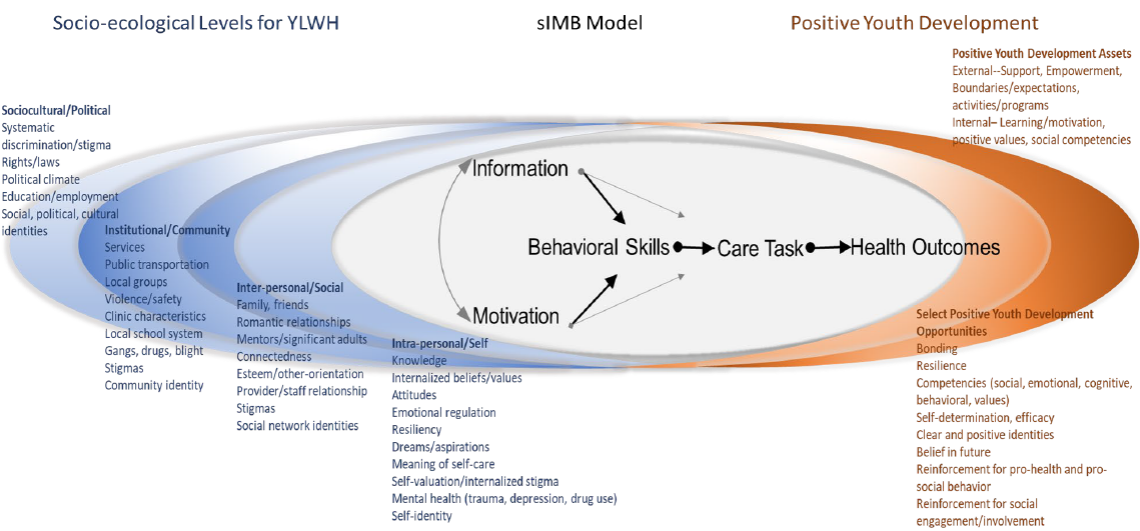
Theoretical underpinning of triggered escalating real-time adherence (TERA) intervention.

The TERA intervention uses EDM to signal coaching opportunities, whereas the implementation of coaching draws heavily from the Information, Motivation, and Behavioral Skills (IMB) model situated within socio-ecological and positive youth development frameworks (Figure 1).

The IMB model of ART adherence [41], which has been used extensively in interventional adherence enhancement research [42,43] and has a developed evidence base in diverse groups of adults [43-45], is used as the basis for understanding specific adherence and nonadherence events. We use the situated application of the Information Motivation Behavioral Skills model (sIMB) [46] to further embed the kinds of knowledge; personal and social consequences of adherence and nonadherence in the context of daily life; and skill sets needed for youth to navigate adherence in the context of self, others, and systems. Tailored understanding of each of the core IMB model constructs as expressed within and between the layers of the socio-ecological model (Figure 1 far left) is further refined with a Positive Youth Development lens (Figure 1 far right), which calls attention to the resources and opportunities unique to adolescents and young adults and is critical in fostering positive awareness of, attitudes toward, and skills in promoting self-care. The coaching intervention uses this synthesized model to guide efforts to engage youth in their current context, within their particular landscape of resources and gaps. Coaches are trained in the social-ecological approach and incorporate these constructs in their discussions focused on factors influencing adherence. Although the models in Figure 1 form the backbone of understanding for how adherence may be optimized or derailed, implementation of coaching discussions are guided by Next Step Counseling (NSC), which draws on Motivational Interviewing (MI). NSC, MI, and specifics for coaching in terms of theory and implementation are detailed in the intervention section. In summary, the aim of the TERA study is to evaluate a novel, evidence-informed triggered, escalating, real-time adherence intervention that leverages coaching and contemporary technology to promote viral suppression among YLWH who have failed on ART.

## Methods

### Trial Design Overview

TERA is a phase 2, randomized, open-label study evaluating the efficacy of the TERA coaching 12- week intervention in YLWH failing ART. A total of 120 YLWH between the ages of 13 and 24 years will be randomized with equal probability to the TERA intervention or continuing SOC, with stratification by age (<18 years vs ≥18 years). At entry, 40 participants (20 from each arm) will be randomly selected to engage in additional in-depth interviews, at study weeks 12 and 48, about their experiences around adherence and self-care as well as their experiences being in the study.

### Study Setting

Clinical research sites within the ATN and the International Maternal, Pediatric, Adolescent AIDS Clinical Trials network in the United States were solicited for interest in participation in ATN 152. A total of 8 clinical research sites are engaged in the trial, including sites in Colorado, Florida, Georgia, Maryland, Michigan, New York, and Tennessee. Clinical research sites differ in the demography of clinic populations, reflecting the specific characteristics of the HIV epidemic within youth in their region. Total anticipated targets for enrollment also vary between clinical research sites, ranging from 5 to 20 youth. All sites are experienced research sites that also operate as clinical care centers for youth. For the TERA study, a minimum of a site-level principal investigator and a study coordinator are required. The University of North Carolina at Chapel Hill (UNC-CH) serves as the single Institutional Review Board (sIRB) and has reviewed and approved the study for study sites.

### Participants

A total of 120 participants will be enrolled. Inclusion and exclusion criteria are listed in Textboxes 1 and 2, respectively.

**Inclusion criteria of participants.**

**Inclusion criteria:**

1. Age: 13 to 24 years.
2. Confirmed HIV positive status: Confirmation of HIV-1 infection as documented in the participant’s medical record by at least 2 criteria.
3. Aware of HIV status: Site staff determined.
4. Viremic: Documented plasma HIV-1 RNA plasma ≥200 copies/ml within 45 days of enrollment visit.
5. On antiretroviral therapy (ART): Prescribed ART at least 24 weeks or more before documented plasma HIV-1 RNA plasma ≥200 copies/ml; prescribed a once-a-day (one or more pills once a day) ART regimen with at least 2 active agents (per clinician judgment or genotype evidence) at enrollment.
6. Language: Able to communicate in spoken and written English.
7. Technology access: Currently has a cellular phone that is able to send and receive short message service text messages.
8. Retention: Willing and able to provide at least 1 additional contact phone number (preferably 2) to be able to contact participant.
9. Consent or assent: Able and willing to provide written informed assent or consent and able to obtain written parental or guardian permission to screen and to enroll in this study.

**Exclusion criteria of participants.**

**Exclusion criteria:**

1. Cognitive capacity: Gross cognitive limitations, acute emotional instability, or medical or mental health illness that would impair the individual’s ability to provide informed consent or interfere with the protocol’s objectives.
2. Concurrent participation: Concurrent participation in interventional studies addressing adherence, unless approved in advance by the study team.
3. Pregnancy: Positive pregnancy test at the time of enrollment; however, if participant becomes pregnant while on study, they may continue the study.
4. Current use of electronic dose monitoring: Currently using or planning to use an electronic dose monitoring and reminder device outside of the study.

### Sample Size

Approximately 54% of youth on ART in the United States are estimated to be virally suppressed [1]. The study was designed to have 85% power to detect a difference of 25% in VLS rates at week 12 between the TERA and SOC arms (assuming a success rate of 60% on SOC). Participants lost to follow-up before week 12 will be classified as failures; hence, no adjustment was made for loss to follow-up in the sample size.

### Randomization

Participants will be randomized to the TERA or SOC study arms with equal probability using Medidata Rave EDC (Electronic Data Capture) software managed by an external data management team. Randomization will be stratified by age to ensure balance in treatment assignments, and institutional balancing will be used to help ensure roughly equal balance in intervention assignments within each site. Once randomized to study arm, the system then randomly determines if the participant is eligible for 1 of the 40 interview slots (20 in each study arm). Those selected are offered participation in in-depth interviews, which will occur at weeks 12 and 48. If refusal rates for interview participation at week 12 are higher than expected, probabilities of selection will be increased during accrual.

### Participant Timeline

Participants are enrolled for 48 weeks of study participation. Those assigned to the intervention condition will receive the intervention during their first 12 weeks of participation, followed by 36 weeks of observation for a total of 48 weeks. All participants are asked to attend clinic study visits at weeks 4, 12, 24, 36, and 48. All participants are asked to dose from an EDM (AdhereTech’s smart bottle) for their full 48 weeks of study participation. The participant experience is depicted in Figure 2. All participants have the “light feature” activated on the smart bottle. This soft blue light band on the bottom of the bottle pulses and then turns to solid light as dose times approach (1 hour before scheduled dose time) and pass (1 hour after dose time) without the bottle being opened. The light feature is a default for all participants throughout the entire period of participation, because early discussions with youth at clinical care sites suggested this feature made the bottle more attractive to use. Although this may inflate adherence, we anticipate habituation over time and any influence would be evenly distributed between the study arms. The feature can be disabled upon request.

### 

**Figure 2:**
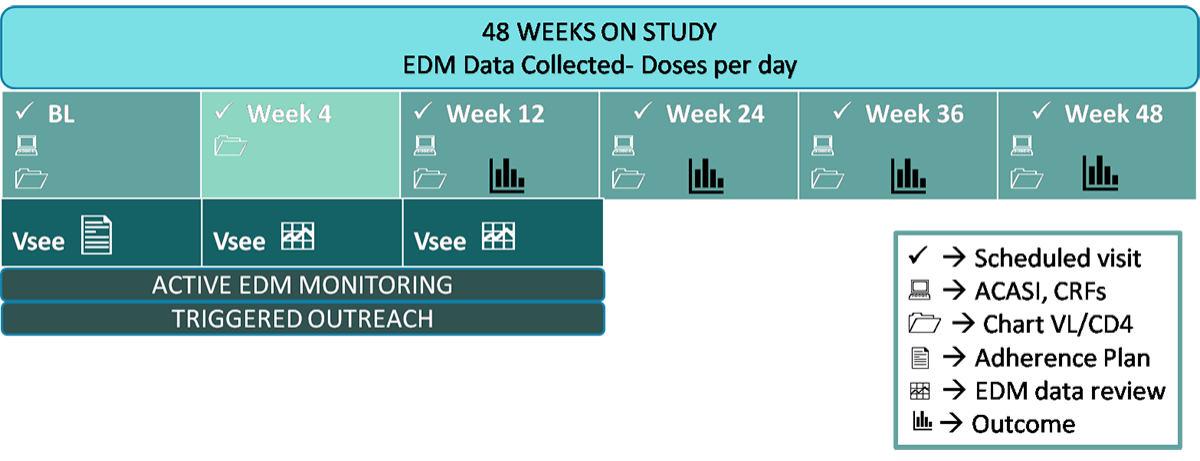
Participant experience.

### 

### 

### 

### Compensation

Compensation will be provided for participants at each study visit. The amount of compensation is determined by the local study site staff, is considered appropriate by the site Institutional Review Board (IRB) and will be confirmed with the sIRB, and will be reflected in the site-specific informed consent form. Recommended incentives include US $75 per visit through week 12 (first 3 study visits); US $40 per visit for weeks 24, 36, and 48; and added incentives for the final week 48 visit (US $100) for a possible total of US $445 over the full 48 weeks. In addition, participants randomly selected for the qualitative interviews receive US $50 per interview for a possible total of US $100. Participants in the intervention are not provided incentives for engagement in intervention sessions or outreach.

### Virtual Youth Advisory Boards

Each participating clinical research site is asked to identify and engage at least 2 YLWH (between the ages of 13 and 24 years) to participate in virtual youth advisory board (vYAB) meetings for the study. These advisory meetings are hosted at the clinic site and use the interactive remote coaching/counseling software program (VSee) with all site youth advisory boards (YABs). Topics discussed include impressions of main challenges for YLWH, vetting ideas about intervention components and study implementation factors, and ensuring that the study and intervention are remaining relevant to the issues germane to YLWH in the regions engaged in the study. vYAB members are reimbursed for their contributions as consultants per meeting attended. Suggested reimbursement is US $50 per meeting, and clinical research sites are provided with food/beverage resources for hosting these meetings. The vYAB meets a minimum of quarterly and as needed.

### Study Conditions–Standard of Care

SOC relative to adherence support will be recorded for each participant during their study participation. In a previous study, we have developed an SOC measure for ART adherence support [47] that follows international recommendations for strategies [48] as well as strategies known to have positive effects [49] in some populations. There are no restrictions on participant SOC adherence support during the study for participants; however, the use of another EDM is not allowed while enrolled.

### Study Conditions–Intervention

Components of the intervention implemented over the 12-week intervention period include the following: (1) remote “face to face” coaching with the assigned adherence coach (delivered via computer connection and through VSee videoconferencing software in a private location at the clinical research site) at baseline and weeks 4 and 12; (2) 1-way, discrete SMS text message (“What’s up?”) at dose time when bottle has not yet been opened for that dosing window (users can disable this upon request); (3) 2-way interactive outreach SMS from the coach if the EDM bottle remains unopened after 1.5 hours post dose time (“What’s the plan?” Reply a. taking now, b. took already, c. taking later, or d. pass) with related coach follow-up; and (4) incorporation of dosing data collected via the electronic dose monitoring into follow-up visits (weeks 4 and 12) to help youth visualize, reflect on, and problem solve around dosing patterns. Tracking and monitoring progress for each of these components occurs in a TERA Implementation Dashboard created for the study. Study implementation material also includes a detailed intervention manual and TERA Implementation Dashboard monitoring guide. Study material will be made available at the completion of the study.

TERA coaches, located at the University of Michigan, are bachelor or graduate level trained staff with experience working with youth. Coaches are not required to match the demographics of participants or be living with HIV. Each coach completes and maintains training on human subjects research ethics and specific training on brief counseling techniques and intervention-specific skills and protocol material. Interactions with participants are intended to draw from problem-solving [50], MI [51], NSC [52,53], Positive Youth Development theory [11], and sIMB [46]. Coaches complete 2 MI [51] workshops and 1 NSC workshop and participate in several simulated sessions to practice techniques and skills before meeting with participants. MI [51] has a long history of use in brief interventions, with promising results on improving adherence for YLWH [54], and NSC [52,53] has been adapted to ART adherence among youth for this study. Intervention material is contained in an intervention manual, with basic steps articulated for each planned face-to-face virtual session, infographics and visuals that are used through the sharing screen function during sessions, and full training on the use of the TERA Implementation Dashboard. Thus, coaches receive training in not only the theories underpinning the intervention approach, basic coaching, MI, and NSC but also on using the technology and software used to deliver the intervention and monitor implementation.

**Figure 3:**
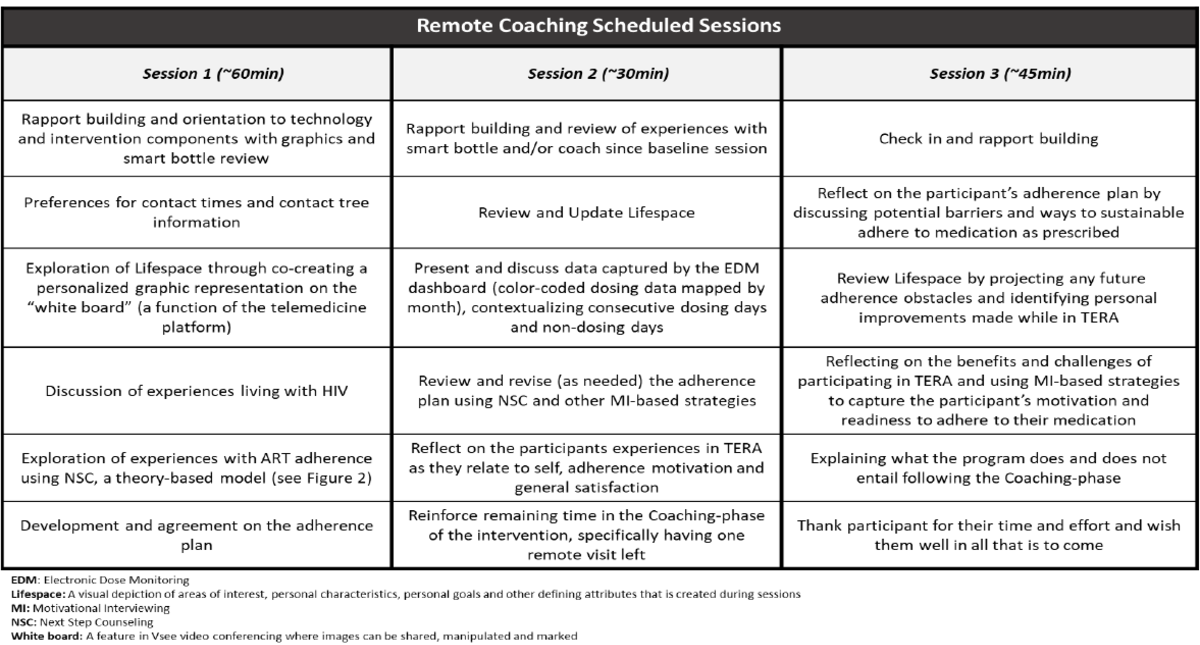
Remote face-to-face coaching sessions.

Youth assigned to the TERA intervention condition at baseline will interact with their assigned adherence coach in a private clinic location through Web-enabled remote coaching (VSee software program). These trained coaches are not part of the clinic team; rather, they are centrally located at the University of Michigan. The general content and flow of each of the 3 planned remote face-to-face sessions are depicted in Figure 3; however, coaches can revise this based on specific needs and context for each participant. Sessions are recorded and transcribed for fidelity checks and supervision. Material created during the session (such as drawing and writing out important factors and people in the participant’s life) on the “white board” feature of VSee is saved for use in subsequent sessions in a secure location.

Information about dose time, preferences, and contacts, in addition to a case note summary of the content of coaching sessions, is entered after the visit in the TERA Implementation Dashboard.

**Figure 4:**
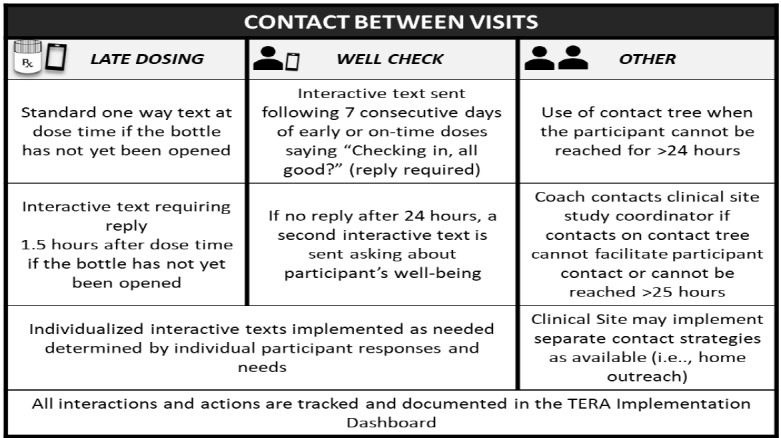
Between session contacts.

In addition to the coaching remote face-to-face interactions, daily dosing is monitored for participants in the intervention condition to identify when text- or phone-based outreach from coaches should be initiated. Between-visit contacts are detailed in Figure 4 and include outreach around late dosing, check-in texts, and other strategies to engage youth who have been difficult to contact. For each outreach around challenges with dosing, the goal is to engage youth before the dose is “missed” to see if dosing may be possible in a supportive manner. A dose is considered missed for intervention purposes only if the EDM remains unopened through to the following day, although we recognize that some participants who have dose times late in the day may in fact still be within an appropriate window to take the dose. Coaches work with participants on a case-by-case basis when conducting follow-up and assisting participants in determining next steps. Each late dose (1.5 hours after dose time without a bottle opening) sends a planning question to the youth’s phone (What’s the plan?) and an alert to the coach or the monitor watching the TERA Implementation Dashboard after hours. The alert creates a “ticket” in the TERA Implementation Dashboard that is followed until closed through successful contact or determined on a case-by-case basis to be resolved.

**Figure 5:**
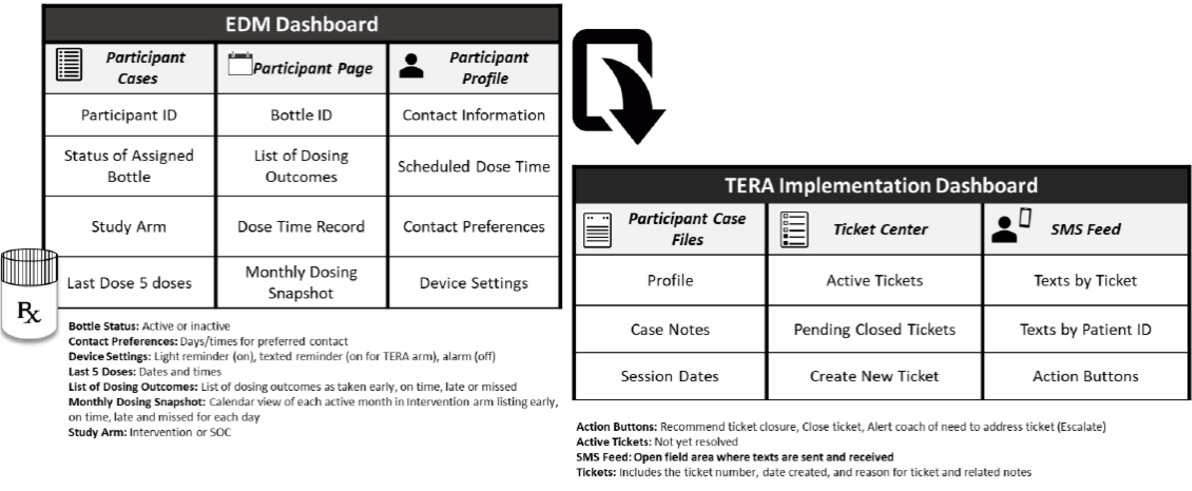
Electronic dose monitoring device dashboard and triggered escalating real-time adherence (TERA) Implementation Dashboard.

Coaches and monitors (research staff who are trained to monitor and triage tickets in the TERA Implementation Dashboard after workday hours and on weekends) track and document implementation of the TERA intervention package in the TERA Implementation Dashboard. The TERA Implementation Dashboard was developed for this study and receives direct input from the EDM device’s proprietary dashboard (AdhereTech). The main components of the EDM manufacturer’s dashboard for the TERA study and the TERA Implementation Dashboard it communicates with are presented in Figure 5. Each secure site plays an important role in the implementation of between-session contacts.

As depicted in Figure 5, the EDM dashboard has daily data specific to whether or not the bottle was opened relative to dose time as well as functionality checks on battery and cell signal strength. The additional details collected and presented in this dashboard allow coaches and monitors to quickly assess dosing patterns as well as the status of dosing (opening events) currently and historically. This dashboard also presents dosing data pictorially with colored marks on each calendar day for *taken early*, *on time*, *late*, or *missed* (no opening in a 12-hour period) dosing. As previously noted, coaches use this graphic in their review of dosing patterns at the week 4 and week 12 remote face-to-face coaching visits.

The detailed tracking of the implementation components of the TERA intervention are collected in the TERA Implementation Dashboard for use in real time to work with youth to prevent missed dosing as well as for use at the end of the study to characterize actual intervention implementation. As can be seen in Figure 5, each participant in the intervention arm has an area for basic details such as contact information and preferences for contact times, case notes to document interactions by date and time (much like an electronic medical record), dates and windows for remote coaching visits, and details surrounding current and historical tickets (virtual medical file). The ticket center lists all open tickets that are in process (active) or pending closure. Tickets are created automatically when the EDM dashboard signals a late dose (1.5 hours after dose time), when participants are automatically texted a check-in message after 7 days of on-time dosing, and can be manually created by coaches for any other type of communication with participants via text or phone. Tickets are also created if a participant texts the coach outside of a communication chain already initiated in response to a specific ticket. Thus, all communications with participants between the scheduled remote face-to-face visits are associated with a ticket number. Texting, both sent and received, is conducted in a specific area of the TERA Implementation Dashboard. All SMS texts are stored in the dashboard. SMS texts sent and received in a communication chain and related case notes are connected to the originating ticket number. This allows data collection and capture of each interaction in the 12-week intervention period. When a participant has been contacted or the issue that started the ticket has otherwise been resolved, supervisors can move tickets from “pending” to “closed,” which provides an added layer of oversight on intervention implementation. In addition to providing a tool for coaches and monitors to implement the intervention, the TERA Intervention Dashboard also serves to collect data (such as number of and content of texts and reasons for or outcomes of outreach attempts) that will be used to characterize intervention implementation.

### Measures

Data collected include responses to Audio Computer-Assisted Self-Interviews (ACASI) scales and items, estimated adherence through collection of “opening events” from the EDM, and chart extracted data. In addition, implementation data are collected to provide costing data. Finally, qualitative interviews are conducted to explore feasibility, acceptability, and overall experiences in the study immediately at the end of the active intervention phase (week 12) and again at the end of participation in the study (week 48). Interview collected data are not used to modify the intervention during TERA but will be used in considering future implementation of this and related intervention approaches.

ACASI data are collected at baseline, week 12, week 24, week 36, and week 48. Table 2 describes each measure used in the ACASI as well as the schedule for data collection and brief description of the measure. The ACASI should take approximately 30 minutes or less to complete.

### Statistical Methods

The primary and secondary objectives of this study are to estimate and compare VLS rates and adherence over 48 weeks between the TERA and SOC arms. Analyses will be intent-to-treat (ITT) using all participants as-randomized to control or intervention arm. Participants lost to follow-up before key time points or with no HIV-1 RNA measurement within allowable windows will be classified as failures at that time point. Categorical outcomes will be summarized using proportions (95% confidence intervals), and continuous outcomes will be summarized using means/medians as appropriate. In adjusted analyses, the number of covariates used in the models will be limited because of the relatively small sample size. Factors that could be associated with the outcome measures and could affect the magnitude of differences between the TERA and SOC arms include age, race, gender, route of HIV infection, years living with HIV, regimen line (eg, first and second), and substance use. A significance level of *P*<.05 will be used with no adjustments for multiple comparisons or interim analyses.

The EDM will provide daily information on adherence in each participant. Overall, 2 outcomes (percentage of days correctly dosed per week and percentage of days dosed within the targeted time frame per week) will be summarized by arm weekly, in 12-week intervals, and between the intervention and postintervention time periods. Differences may be largest during the initial 12 weeks, as that is when the intervention is administered, with differences waning over time. To address possible informative censoring induced by losses to follow-up, analyses will include (1) available data and (2) imputing weekly adherence of 0% if a participant is lost to follow-up.

To characterize TERA implementation, ability to enroll to the study, drop-out rates by week 12 and throughout the study, numbers of participants escalating to different alerts and outreach at least once, numbers of alerts per participant (TERA arm only), and themes from qualitative interview content related to experiences in the intervention will be summarized.

### Ethics

The ATN Coordinating Center at UNC-CH provides sIRB approval, guidance, and monitoring. All clinical research sites ceded regulatory oversight to the IRB at UNC-CH. All sites adapt an sIRB approved consent template to meet the specific requirements of their site. Waivers of parental consent for those under the age of 18 years were considered by all sites and adopted by those with local regulatory approval.

### Study and Data Monitoring

On-site monitors from the ATN Coordinating Center will review a selected portion of the individual participant records, including assent/consent forms, case report forms (CRFs), and supporting source documentation to ensure the protection of study participants, compliance with the protocol, and accuracy and completeness of records. Regulatory files, as required, will also be inspected to ensure that regulatory requirements are being followed.

The Protocol Team will review accrual, retention, and data quality on monthly team calls, with data combined across study arms. An independent Study Monitoring Committee (SMC) will review the study at scheduled, planned points to monitor participant safety as well as data integrity. At each review, the SMC may recommend that the study proceed as currently designed, proceed with design modifications, or be discontinued. The SMC may also provide specific operational recommendations to help address any study implementation challenges identified during their reviews. Untoward events will be recorded by the site and reported to the ATN Coordinating Center, study team, and the sIRB.

**Table 2:** List of study measures and collection time points.

Measures^a^	Collection method	Study visit week					Description
		BL^b^	12	24	36	48	
Adherence support during participation	ACASI^c^	─^d^	X^e^	─	─	X	Checklist of receipt of specific kinds of support during the first 12 weeks and at week 48
Information Motivation Behavior Skills ART^f^ Adherence Questionnaire [55,56]	ACASI	X	X	X	X	X	Measure of adherence barriers identified by the Information, Motivation, Behavioral Skills Model of adherence
The HIV Adherence Self-Efficacy Scale [57]	ACASI	X	X	X	X	X	Measures self-efficacy for adherence to HIV treatment plans, including, but not limited to, taking HIV medications
Adolescent Decision-Making Questionnaire (ADMQ) [58]	ACASI	X	X	X	X	X	Revised version of the ADMQ that measures decision-making patterns in adolescence: avoidance, self-confidence, panic, and impulsive/thoughtless
Center for Epidemiological Studies Depression Scale (CESD-10) [59]	ACASI	X	─	─	─	X	Self-reported 10-item screener for depressed mood in respondents
Demographics	ACASI	X	─	─	─	─	Study developed and ATN-harmonized items assessing sociodemographic characteristics
Emotional Regulation Questionnaire [60]	ACASI	X	X	X	X	X	10-items scale designed to measure cognitive reappraisal and expressive/suppressive regulation
EuroQOL Five Dimensions Questionnaire for Youth (EQ-5D-Y) (overall health status) [61]	ACASI	X	X	X	─	X	Standardized measure of overall health status: mobility, looking after myself, doing usual activities, having pain/discomfort, and sad or happy, using a visual analog scale
HIV cascade measure (ATN Coordinating Center)	ACASI	X	─	─	─	─	ATN-harmonized items related to engagement in HIV-related care
HIV stigma mechanisms [62]	ACASI	X	X	X	X	X	Stigma framework including measures of internalized, anticipated, and enacted HIV stigma
Life Events Survey	ACASI	X	X	─	─	X	Study-adapted measure of significant or traumatic life events
Satisfaction scale (developed for study)	ACASI	─	X	─	─	X	Study-developed measure of participants’ satisfaction with the TERA^g^ intervention
Self-reported adherence [63]	ACASI	X	X	X	X	X	3-items: *doses taken* (0 to 30), *frequency* of doses taken in last 30 days, and *rating* of how good of job taking medications
Sex behavior	ACASI	X	X	─	─	X	Brief item set to assess rates of condomless sex
Social Support Scale (Medical Outcomes Study ) [64]	ACASI	X	X	X	X	X	Overall functional social support and emotional/information and tangible, affectionate, and positive social interaction support
Substance use (Alcohol, Smoking and Substance Involvement Screening Test) [65]	ACASI	X	X	─	─	X	Problem or risky use of tobacco, alcohol, cannabis, cocaine, amphetamine-type stimulants, sedatives, hallucinogens, inhalants, opioids, and “other drugs” that do not fall into the previous categories
Adherence support services utilization checklist	Participant visits and interviews	X	X	X	X	X	Study-developed checklist completed by study staff to document standard of care adherence support services received by participant
Medical history	Chart abstraction	X	X	X	X	X	Date of HIV diagnosis, route of HIV transmission, previous ART regimens, opportunistic infections since diagnosis, comorbidities, and concomitant medications
Qualitative interviews	Remote (VSee) interview	X	─	─	─	X	Main themes youth report for adherence support needed, received, and valued

^a^EDM adherence data are collected throughout study participation, that is, from baseline to the 48-week visit. Chart-abstracted data for all HIV-VL and CD4 tests conducted while on study will be extracted; VL test results at baseline, week 12, and week 48 are required and resourced by the study if clinical care did not involve a VL test at those visits.

^b^Baseline.

^c^ACASI: Audio Computer-Assisted Self-Interviews.

^d^Not included in visit.

^e^Conducted or included in the visit.

^f^ART: antiretroviral therapy.

^g^TERA: triggered escalating real-time adherence.

### 

### 

### 

## Results

To date, all clinical research sites have ceded to the UNC-CH sIRB and are in the process of opening for enrollment. First enrollments occurred in April 2018 with a planned 12-month period to reach the target enrollment of 120 youth. Original timelines anticipated enrollment to begin in January 2018, allowing enrollment to end in January 2019. An adjusted timeline, allowing enrollment to extend to August 2019, would allow completion of data collection by August 2020 and dissemination of results in the second quarter of 2021. Presently, 8 sites are open to enrollment and 46 youth have been enrolled in the study.

## Discussion

### Summary

The TERA study will contribute to the developing evidence base focused on better understanding the dynamics that influence ART adherence in youth. This study advances the number of options for highly generalizable strategies to optimize adherence among YLWH known to have struggled with ART in the past. As the approach uses centralized coaches and electronic dose monitoring that are separate from the clinical care team and related resources, if effective, the program could be brought to scale as a support service offered to youth throughout the United States resourced by insurers or stakeholders invested in optimal adherence. Interventions that are matched to maturational issues and demands of youth are critically needed [7,37]. TERA results should contribute to advancing both the science and practice of providing optimized support to YLWH.

### Limitations

There are several limitations that are important to consider. The EDM device tracks opening events and not consumption of medications. Days with opening events cannot assure consumption, and days passing without opening events cannot confirm nonconsumption. We do note that EDM data do perform well as a proxy for adherence. Evaluations of EDM-collected opening events do provide evidence for a consistent association with viral load and when compared with other measurement approaches (self-report, pill-count, and pharmacy refill) tend to demonstrate higher associations with clinical outcomes [66-68]. Another assumption of the TERA intervention is that failure to achieve and/or sustain viral suppression is secondary to adherence problems. Working with youth in the United States where resistance testing is a part of clinical care, especially with documented treatment failure, we feel this may be a reasonable assumption. In addition, among youth with perinatal infection, a recent study has suggested that drug resistance is less frequently the cause of virologic failure to new ART regimens than nonadherence [69,70]. Clearly, one would not expect the TERA intervention to be effective in assisting youth with viral suppression if the root cause for failure is resistance. In addition, the intervention uses SMS texting as a method for contacting youth. If participants’ cell phones become discontinued or numbers are changed, lost, or replaced, there is no way of knowing on the basis of texting. To mitigate this, coaches include specific discussions on what to do in these situations with each intervention arm participant at the initial and subsequent remote coaching sessions. In addition, coach procedures allow contacting site coordinators to investigate this in the event that they have had several days without participant reply or response to outreach.

### Conclusions

EDM, real-time triggered interventions, and interactive and real-person phone-based outreach with the use of a contact-tree are all novel components to adherence support that promise high impact. The existing evidence base will be leveraged to create a high-intensity, responsive, time-limited intervention approach. Although texting has a strong evidence base for adults [26,28,41], use with youth, although intuitively appealing given the widespread use of texting, remains supported largely only by pilot studies [31]. Similarly, phone-based problem-solving discussion with adherence coaches has preliminary evidence [34] demonstrated in a pilot study. This study leverages the wealth of pilot evidence to create an intervention approach with demonstrated promise but not yet rigorously evaluated. Of particular interest, our goal is to mesh together an evidence-informed approach that can also be generalizable. Given that sites and clinics working with youth will have limitations in resources, we adapted interventions implemented over an extended period to a discreet, intensive approach implemented over a 12-week period and intensified in response to delayed dosing. This creates a more generalizable program as resources required are similarly time-limited. The key pieces that make up the TERA intervention are largely in place; YLWH overwhelmingly have cell phones and clinic team members already use or will be trained in problem-solving. A system for sending and receiving texts can be automated, with costs allocated toward building the system and minor costs for maintenance of system. If the intervention is effective, it could have an immediate impact on care services provided to YLWH failing ART and future applications to other points in the continuum of HIV prevention and care that depend on youth adhering to the applicable interventions.

## Abbreviations

ACASI: Audio Computer-Assisted Self-Interview
ADMQ: Adolescent Decision-Making Questionnaire
ART: antiretroviral therapy
ATN: Adolescent Medicine Trials Network for HIV/AIDS Interventions
CRF: case report forms
EDM: electronic dose monitoring
EQ-5D-Y: EuroQol 5 dimensions Questionnaire-Youth
IMB: Information, Motivation, Behavioral Skills Model
IRB: Institutional Review Board
MI: motivational interviewing
NSC: Next Step Counseling
SDAC: statistical data analysis center
sIMB: situated application of the Information Motivation Behavioral Skills model
sIRB: single Institutional Review Board
SMC: Study Monitoring Committee
SMS: short message service
SOC: standard of care
TERA: triggered escalating real-time adherence
UNC-CH: The University of North Carolina at Chapel Hill
VLS: viral suppression
vYAB: virtual youth advisory board
YAB: youth advisory board
YLWH: youth living with HIV
